# 

**Published:** 1908-05

**Authors:** 


### BOOKS RECEIVED.

International Clinics. A Quarterly of Illustrated Clinical Lectures and especially prepared articles on Treatment, Medicine, Surgery, Neurology, Pediatrics, Obstetrics, Gynecology, Orthopedics, Pathology, Dermatology, Ophthalmology, Otology, Rhinology, Laryngology, Hygiene and other topics of interest to students and practitioners. Edited by W. T. Longcope, M.D., Volume I., eighteenth series. Philadelphia and London: J. B. Lippincott Co. 1908. (Cloth, $2.00.)

Nervous and Mental Diseases. For Students and Practitioners. By Charles S. Potts, M.D., Professor of Neurology in the Medico-Chirurgical College of Philadelphia. Second edition. 12mo, 570 pages, with 133 engravings and 9 full-page plates. Lea & Febiger, Philadelphia and New York. (Cloth, $2.50 net.)

Bier's Hyperemic Treatment in Surgery, Medicine and the Specialties. By Willie Meyer, M.D., Professor of Surgery at the New York Post-Graduate Medical School and Hospital. And Professor Dr. Victor Schmieden, Assistant to Professor Bier, University of Berlin, Germany. Octavo, pp. 209. Illustrated. Philadelphia and London: W. B. Saunders Co. 1908. (Cloth, $3.00.)

An Index of Treatment by various writers. Edited by Robert Hutchison, M.D., F.R.C.P., Physician to London Hospital, and H. Stansfield Collier, F.R.C.S., Surgeon to St. Mary's Hospital, London. Revised by Warren Coleman, M.D., Professor of Clinical Medicine in Cornell University Medical College. Octavo, pp. 888. New York: William Wood & Co., 1908. (Cloth, $6.00.)

A Manual of the Diseases of Infants and Children. By John Ruhrah, M.D., Clinical Professor of Diseases of Children in the College of Physicians and Surgeons, Baltimore. Second edition. 12mo, pp. 423. Philadelphia and London: W. B. Saunders Co. 1908. (Limp leather, $2.00.)

Report of the Commission of 1906 to Investigate the Condition of the Blind in the State of New York. F. Park Lewis, M.D., Buffalo, President. Transmitted to the legislature April 10, 1907.

American Practice of Surgery. A complete System of the Science and Art of Surgery by representative surgeons of the United States and Canada. Edited by Joseph D. Bryant, M.D., and Albert H. Buck, M.D., New York. Complete in eight volumes. Vol. IV. Imperial octavo, pp. 1010. Profusely illustrated. New York: William Wood & Company. 1907. (Price, cloth, $7.00).

